# Effects of Synthesis Parameters on Structure and Antimicrobial Properties of Bacterial Cellulose/Hydroxyapatite/TiO_2_ Polymer–Ceramic Composite Material

**DOI:** 10.3390/polym16040470

**Published:** 2024-02-07

**Authors:** Aleksandra Sknepnek, Suzana Filipović, Vladimir B. Pavlović, Nemanja Mirković, Dunja Miletić, Jelena Gržetić, Miljana Mirković

**Affiliations:** 1 Faculty of Agriculture, University of Belgrade, Nemanjina 6, 11080 Belgrade, Serbia; vlaver@agrif.bg.ac.rs (V.B.P.); nemanja.mirkovic@agrif.bg.ac.rs (N.M.); dunja.miletic@agrif.bg.ac.rs (D.M.); 2 Institute of Technical Sciences of SASA, Kneza Mihaila 35/IV, 11000 Belgrade, Serbia; suzana.filipovic@itn.sanu.ac.rs; 3 Department for Materials and Protection, Military Technical Institute, Ratka Resanovića 1, 11030 Belgrade, Serbia; jrusmirovic@tmf.bg.ac.rs; 4 Department of Materials, “VINČA” Institute of Nuclear Sciences—National Institute of the Republic of Serbia, University of Belgrade, Mike Petrovića Alasa 12—14, 11351 Belgrade, Serbia; miljanam@vin.bg.ac.rs

**Keywords:** bacterial cellulose, *Komagataetibacter rhaeticus*, hydroxyapatite, titanium dioxide, polymer–ceramic material, antimicrobial activity

## Abstract

Bacterial cellulose (BC) is a highly pure polysaccharide biopolymer that can be produced by various bacterial genera. Even though BC lacks functional properties, its porosity, three-dimensional network, and high specific surface area make it a suitable carrier for functional composite materials. In the present study, BC-producing bacteria were isolated from kombucha beverage and identified using a molecular method. Two sets of the BC hydrogels were produced in static conditions after four and seven days. Afterwards, two different synthesis pathways were applied for BC functionalization. The first method implied the incorporation of previously synthesized HAp/TiO_2_ nanocomposite using an immersion technique, while the second method included the functionalization of BC during the synthesis of HAp/TiO_2_ nanocomposite in the reaction mixture. The primary goal was to find the best method to obtain the functionalized material. Physicochemical and microstructural properties were analyzed by SEM, EDS, FTIR, and XRD methods. Further properties were examined by tensile test and thermogravimetric analysis, and antimicrobial activity was assessed by a total plate count assay. The results showed that HAp/TiO_2_ was successfully incorporated into the produced BC hydrogels using both methods. The applied methods of incorporation influenced the differences in morphology, phase distribution, mechanical and thermal properties, and antimicrobial activity against *Staphylococcus aureus* (ATCC 25923), *Escherichia coli* (ATCC 25922), *Proteus mirabilis* (ATCC 12453), and *Candida albicans* (ATCC 10231). Composite material can be recommended for further development and application in environments that are suitable for diseases spreading.

## 1. Introduction

The resistance of microorganisms to antibiotics is rapidly increasing, which is leading to high mortality levels within the human population. Current estimates indicate annual rates of 33,000 deaths in the European Union and 700,000 deaths globally per annum, with a projected significant increase of up to 10 million by the year 2050 caused by multidrug-resistant microorganisms. Therefore, scientific efforts are focused on prevention strategies, innovative solutions in the inhibition of pathogens’ proliferation, and their spreading in nature and society [1,2]. The physicochemical and functional properties of metal oxide nanoparticles have been intensively investigated in the past decades for application as antimicrobial agents, and it was found that they express strong ability to act against bacteria. Due to their action on bacterial cell walls, it is assumed that microbial cells will not develop resistance towards metal oxide particles over time [3,4]. Among them is titanium dioxide (TiO_2_), which, as a semiconducting transition metal oxide material, stands out for its high biocide potential. This ability originates from the photocatalytic properties and generation of reactive oxygen species (ROS). ROS disable the antioxidant defense system of microorganisms and cause the death of cells due to damage of the cell membrane and further intracellular antimicrobial effect on cell’s DNA and proteins [5]. TiO_2_-based materials have found an application in various fields, such as white color in paints, papers, plastics, or ceramics, but also in air and water purification systems, for sterilization, or UV protection. Also, these materials have been very useful against pathogens when applied as a coating or as antimicrobial paint on hospital touch surfaces [2]. However, the usage of TiO_2_ in organic materials, paints, or plastics is limited due to their photocatalytic activity and consequent degradation of these materials. To protect the carrier material in the composite from photocatalytic degradation, TiO_2_ needs to be blended with other materials such as hydroxyapatite (HAp, Ca_10_(PO_4_)_6_(OH)_2_). As has been previously found, it is possible to protect acrylic resin from photocatalytic degradation when HAp is incorporated into the composite material with TiO_2_ [6]. HAp material has found a variety of applications, such as in the production of chromatographic columns due to its ability to adsorb proteins, or in respirators to adsorb pollen, as well as bacteria in hand creams. In the research of Janovák et al. [7], the synergistic effect of HAp/TiO_2_ composite was proved to occurs due to a bacterial adhesive nature to the HAp and improved TiO_2_ surface accessibility. Nonami et al. [6] developed ceramic composite surfaces made from TiO_2_ covered by HAp, where HAp adsorbs bacteria and TiO_2_ is responsible for the decomposition of bacterial cells.

The most abundant and widely applied natural polysaccharide, cellulose, can be synthesized by plants, microorganisms, and some animals [8]. The cellulose in plants is one of the major components in lignocellulosic biomass besides aromatic-rich polymer lignin and carbohydrate polymer hemicellulose that are considered impurities and thus demand removal by using complex mechanical, chemical, and enzymatic purification treatments. However, cellulose produced by bacteria is pure and free of by-products, making it a highly appealing material that requires a less aggressive and environmentally friendly purification process. Overall, less energy consumption is needed for this processing step, while the waste effluents are less toxic compared to pulping and bleaching steps during plant-based cellulose production [9,10,11]. BC is dominantly produced by several bacterial genera: *Agrobacterium*, *Aerobacter*, *Rhizobium*, *Sarcina*, *Azotobacter*, *Salmonella, Pseudomonas*, and *Bacillus* genera, as well as by the *Acetobacteriaceae* family [12,13]. In the latest family, the genus *Komagataeibacter* is recognized as the major BC producer. Synthesized BC has a crystal structure that is chemically the same as plant cellulose and represents a linear β-glucan biopolymer with several thousand polymerization degrees of 1,4-linked glucopyranosyl residues [10,11]. Nevertheless, the advantages of BC are numerous: thinner threads, better crystallinity, higher mechanical strength, and purity. Although BC-based materials lack functional properties, their porous structure and three-dimensional network of nanofibers with a high specific surface area are suitable as a carrier of antimicrobials or other agents in the production of functional composite materials [14]. Thus, cellulose-based nanocomposites have been the subject of many research articles in the last decade [14,15,16,17]. Namely, nanoparticles made of carbon quantum dots and TiO_2_ added in BC expressed antibacterial properties against *S. aureus* [3]. Porous microsphere made from cellulose nanocrystals, alginate, and ξ-polylysine inhibited activities of *E. coli*, *Pseudomonas aeruginosa*, and *S. aureus* and was recommended for wound healing [18]. Xuchao et al. [19] developed a nanocomposite scaffold by incorporating TiO_2_ into the composite made of plant cellulose and HAp using a freeze-drying technique. The scaffold with the highest percentages (wt.%) of HAp and TiO_2_ had the highest capacity to remove lead ions as heavy metals from aqueous environments.

In this research, cellulose-producing acetic acid bacteria (AAB) were isolated from kombucha beverage to be applied in the development of BC hydrogels as a matrix for the incorporation of ceramic HAp/TiO_2_ composites, with the aim of acquiring the antimicrobial properties of BC/HAp/TiO_2_. In order to achieve antibacterial and antifungal properties of BC and to avoid destruction of the organic component at the same time, two synthesis methods for incorporation of the HAp/TiO_2_ ceramic component were applied. For the first method, ceramic composite was synthesized prior to incorporating into BC hydrogel. The second approach was to synthesize HAp/TiO_2_ composite in the presence of BC hydrogel polymer in a synthesis solution. The effect of different synthesis routes on the morphology, crystal structure, lattice vibrations, and antimicrobial properties of the obtained hybrid materials was investigated. To the best of our knowledge, this is the first time that HAp/TiO_2_ ceramic filler was incorporated into BC polymer concerning the best antimicrobial properties of novel hybrid composite, with well-defined structural, morphological, mechanical, and thermal characteristics.

## 2. Materials and Methods

### 2.1. Isolation and Molecular Identification of Cellulose-Producing Bacteria

To find the best cellulose-producing bacterial strain, AAB were isolated from black tea kombucha broth on the fifth day of fermentation according to the method of Amoa-Awua et al. [20]. To isolate AAB, kombucha was homogenized, and 1 mL of an appropriate dilution of the kombucha broth was transferred into a Petri dish. Afterwards, sterile YPM agar (yeast extract 5 g/L, peptone 3 g/L, mannitol 25 g/L, agar 12 g/L) supplemented with cycloheximide (10 mg/mL, AppliChem GmbH, Darmstadt, Germany, dissolved in 50% ethanol) and penicillin (20 mg/L, Bioanalyse, Ankara, Turkey, prepared as a 0.25% solution) was poured. Incubation was performed at 30 °C for 5 days. Morphologically different colonies were sub-cultured on the YPM agar plates until pure cultures were obtained. The purity of the bacterial cultures was confirmed under light microscopy (Olympus, Tokyo, Japan) after Gram staining. To find the strain with the highest yield, which will be further identified, YPM broth (yeast extract 5 g/L, peptone 3 g/L, mannitol 25 g/L) was applied to cultivate cellulose-producing bacteria. The best cellulose-producing bacterial strain was stored in the culture collection of the Department of Industrial Microbiology, Faculty of Agriculture, University of Belgrade, at −80 °C, in YPM broth with the addition of glycerol (20% *v*/*v*).

For the molecular identification of bacterial strain, total DNA from selected isolate was obtained by the method of Hopwood et al. [21]. Pure culture of bacteria from YPM agar was seeded in YPM broth. Cultivation was performed on an incubator shaker (SI 600R, Jeio Tech co., Ltd., Daejeon, Republic of Korea) at 180 rpm at 25 °C until the logarithmic growth phase was reached, which was determined spectrophotometrically (BioSpectrometer, Eppendorf, Hamburg, Germany) when an absorbance of 0.6–0.8 was achieved at a wavelength of 600 nm. Bacterial cell broth (10 mL) was centrifuged at 4500 rpm for 10 min (Eppendorf 5804R). The resulting cell pellet was resuspended in 500 μL TEN buffer (50 mM Tris-HCl pH 8; 10 mM EDTA; pH 8; 50 mM NaCl) and centrifuged at 13,000 rpm for 1 min (5415, Eppendorf). Taq DNA polymerase (Kapa Biosystems Inc., Boston, MA, USA) was used to amplify the 16S rRNA gene using a GeneAmp PCR system (Kyratec, Wembley, Australia) and specific primers P1 16S Fw (5′-GAGAGTTTGATCCTGGC-3′) and P2 16S Rev (5′-AGGAGGTGATCCAGCCG-3′) [22]. For visualization of PCR products, 1% agarose gel at a constant voltage of 80 V was used. PCR products were purified using a PCR product purification kit (FastGene Gel/PCR Extraction Kit, Nippon Genetics Europe GmbH, Düren, Germany). Finally, pure PCR products were sequenced by the Macrogen Sequencing Service (Macrogen Europe, Amsterdam, The Netherlands) and analyzed using the BLAST algorithm for nucleotide sequences [23].

### 2.2. Bacterial Cellulose Synthesis, Functionalization Process, and Yield

BC synthesis by the selected AAB strain was performed in YPM broth (yeast extract 5 g/L, peptone 3 g/L, mannitol 25 g/L) for 3 days at 25 °C to prepare the inoculum. Further, the inoculum (5 mL) was transferred to 300 mL Erlenmeyer flasks containing 50 mL of YPM broth. The cultivation was conducted at 25 °C in static conditions. Two sets of hydrogels were prepared after 4 and 7 days of growth. Formed BC hydrogels were removed, rinsed with distilled water, and purified by boiling in 0.1 M NaOH at 90 °C for 2 h. The obtained pellicles were washed in distilled water until pH 7 was reached. BC hydrogels were stored at 4 °C in distilled water until further use.

**Figure 1:**
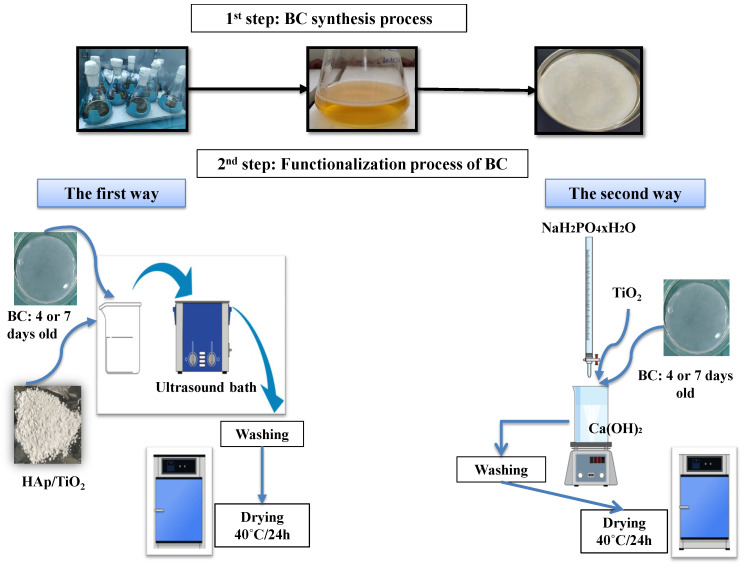
Synthesis and functionalization schemes of two methods for the development of BC/HAp/TiO_2_ composites.

The functionalization of BC hydrogels by HAp/TiO_2_ was performed by two synthesis methods (I and II), explained in detail below and shown in Figure 1.

(I) Synthesis method I:
  First, the warm solution (80 °C) of 0.5 M Ca(OH)_2_ (Centrohem, Stara Pazova, Serbia, extra pure > 96%) was stirred with a magnetic stirrer and titrated with 0.3 M solutions of NaH_2_PO_4_ × H_2_O (≥99.0%, Sigma Aldrich, St. Louis, Missouri, USA, p.a.). At the beginning of the titration process, TiO_2_ (99.5%, p.a. Sigma Aldrich) was added. The pH value was adjusted by NH_4_OH (Centrohem, p.a., 25%) to reach pH 12. The formed HAp/TiO_2_ slurry was rinsed four times with distilled water and once with 96% ethanol. The powder was dried at 40 °C and calcined at 300 °C for 6 h in the air in a tube furnace (Protherm PTF16/75/450, Ankara, Turkey) using the following temperature program: heating to 300 °C at a rate of 10 °C/min, dwell time at 300 °C for 6 h, and cooling to room temperature naturally. In the second step, BC hydrogels were immersed into 50 mL of distilled water containing 75 mg previously added HAp/TiO_2_. The mixture was treated with ultrasonic cleaner (UCP-02, JeioTech co., Ltd., Daejeon, Republic of Korea) for 45 min, rinsed with distilled water and 96% *v*/*v* ethanol, and oven dried at 40 °C. The BC/HAp/TiO_2_ samples obtained by the first functionalization process of BC hydrogels were labeled as 4I and 7I according to days of BC growing.
(II) Synthesis method II:
  To prepare BC/HAp/TiO_2_ by the second method, TiO_2_ was added in the Ca(OH)_2_ solution at the beginning of the process. During the titration of Ca(OH)_2_ solution with NaH_2_PO_4_ on magnetic stirring, BC hydrogel was immersed into the reaction mixture and kept for 1 h in it. Afterwards, the composites were rinsed four times with distilled water and once with 96% *v*/*v* ethanol, followed by oven drying at 40 °C. The obtained BC/HAp/TiO_2_ composites were marked as 4II and 7II.

Control samples of BC hydrogels were obtained after 4 or 7 days of synthesis, without functionalization, and were denoted as 4K and 7K, respectively.

All prepared samples were weighed after the drying process, and the yields were given in mg/50 mL. The amount of incorporated ceramic material in BC was calculated by subtracting the weight of BC/HAp/TiO_2_ composite and the weight of pure BC.

### 2.3. Characterization of Bacterial Cellulose and Bacterial Cellulose-Based Composites

Morphology and elementary composition of the composites and corresponding control samples were analyzed by scanning electron microscopy (SEM) and energy-dispersive X-ray (EDS) techniques. Samples were first coated with gold for 100 sec at 30 mA on the Bal-tec device SCD 005 Sputter coater, (Schalksmühle, Germany). The recording was performed on the JEOL JSM-6390LV scanning electron microscope (JEOL USA, Inc., Peabody, MA, USA) coupled with electron dispersive spectroscopy (EDS; Oxford Instruments X-MaxN, High Wycombe, UK).

The degree of crystallinity of the polymers and phase analysis were performed by X-ray diffraction (XRD). The Ultima IV Rigaku diffractometer equipped with a copper (Cu) X-ray tube anode, CuKα_1,2_ radiation, was used. The samples were cut into 1 × 1 cm^2^ squares and placed on a monocrystalline silicon carrier. The recording was performed under a voltage of 40.0 kV and a generator current of 40.0 mA. The applied recording range was from 5 to 60° 2*θ*, with a step of 0.02° and a recording speed of 5°/min using a D/TeX Ultra-fast detector, Rigaku, Tokyo, Japan. All the measurements were performed at room temperature. For phase analysis and identification, PDXL2 (Ver. 2.8.4.0) software, Rigaku, Tokyo, Japan was used equipped with the ICDD crystallographic database. Selected card numbers were used for phase identification: titanium oxide (01-075-3841), hydroxyapatite (01-073-8417), cellulose I alpha (00-056-1719), cellulose I beta (00-056-1718), and anatase (01-070-6826). The degree of crystallinity can be explained as the ratio of the sum of the deconvoluted crystalline part over the sum of the crystalline and the amorphous deconvoluted parts. The degree of crystallinity was calculated by deconvolution in Gaussian curves and was performed with 3 curves for the crystalline part and 3 curves for the amorphous part. The degree of crystallinity of 4K and 7K samples is calculated as shown in Equation (1):

*Xc* = [*Ac*/(*Aa* + *Ac*)] × 100 (1)

*Ac* is the area of crystalline peaks from the diffraction pattern; *Aa* is the area of amorphous peaks of diffraction.

Fourier transform infrared (FT-IR) spectra were recorded on a Thermo Scientific™ Nicolet™ iS™10 FT-IR Spectrometer (Waltham, MA, USA), equipped with attenuated total reflectance (ATR) accessory. The ATR/FT-IR measurements were performed in the wavenumber region of 400–4000 cm^−1^, with a resolution of 4 cm^−1^, and at room temperature.

Mechanical characterization was determined by the Instron 1122 Universal Testing Instrument (Norwood, MA, USA), by the determination of tensile properties of the control and the functionalized BC-based composites. The standard samples of a rectangular bar shape (44 × 10 × 0.1 mm) were tested at 20 °C with a force loading rate of 1.0 mm/min^−1^. The tensile strength, σ_m_, elongation at break, ε_m_, and Young’s modulus, *E*, were determined to investigate the effect of the applied BC synthesis/modification methods on the structural integrity of the composite.

The thermogravimetric analysis (TGA) of BC and BC-based composites was performed using Setsys SETARAM Instrumentation (Caluire, France) within a temperature range between 25 °C and 600 °C. The experiments involved applying heating rates of 5 °C min^−1^. Subsequent analyses were conducted using the ThermV v0.2 software package [24].

In order to investigate the wettability of BC-based composites, a Delta Smart 5.0 MP Pro, optical microscope (Mińsk Mazowiecki, Poland) was used. The contact angle was determined using Image Proplus 6.0 image analysis software (Media Cybernetics, Rockville, MD, USA) for image analysis.

### 2.4. Antimicrobial Activity Assay

The antimicrobial activity of the obtained BC-based samples was tested against Gram-positive bacteria *Staphylococcus aureus* (ATCC 25923), Gram-negative bacteria *Escherichia coli* (ATCC 25922), as well as *Proteus mirabilis* (ATCC 12453) and pathogenic yeast *Candida albicans* (ATCC 10231) by a total plate count assay. Prior to the investigation, 30 mg of each BC-based sample was sterilized in an autoclave at 121 °C for 15 min. The tested bacteria were prepared by cultivation on Müeller Hinton agar (MHA, HiMedia, Thane, Maharashtra, India) for 24 h at 37 °C. At the same time, yeast was cultivated on Malt agar (MA, Torlak, Belgrade, Serbia) for 24 h at 30 °C. Obtained colonies were used for the preparation of the inoculum. Suspensions of the microorganisms were prepared in Müeller Hinton broth (MHB, HiMedia) for the bacteria or in Malt broth (MB, Torlak) for the yeast. The initial concentration of microorganisms was adjusted to ~10^5^ colony-forming units per mL (CFU/mL) using a DEN-1 McFarland densitometer (Biosan, Riga, Latvia). The number of viable cells of inoculums was determined by a total plate count assay and contained 4.43 log_10_ CFU/mL, 5.02 log_10_ CFU/mL, 5.01 log_10_ CFU/mL, and 5.64 log_10_ CFU/mL for *C. albicans*, *S. aureus*, *E. coli*, and *P. mirabilis*, respectively. The ability of the tested samples to reduce the number of test microorganisms was performed by adding the suspension of microorganisms (1 mL) to each sample, followed by incubation for 24 h at 37 °C or 30 °C for bacteria and yeast, respectively. In a positive control, microbial broths without samples were prepared and treated similarly. The antimicrobial activity of the BC-based samples was determined by counting the number of remaining viable cells (colony forming units—CFU) after preparing serial dilutions in normal saline and plating at MHA or MA for bacteria and yeast, respectively. The results are shown as a number of viable cells in log_10_ CFU/mL, and their reduction in log_10_ CFU/mL was calculated as the difference in log_10_ CFU/mL between the positive control and samples with BC and BC composites. Results are presented as the mean value ± standard deviation for the measurements that were performed in triplicate. Statistical analysis was performed using Origin Pro 9.0. Software. Tukey’s honestly significant difference (HSD) test was applied to determine the statistical significance (*p* < 0.05) between different values.

## 3. Results and Discussion

### 3.1. Identification of the Cellulose-Producing Isolate and Yields of BCs and Composites

AAB are known as good producers of BC, especially species that belong to *Acetobacter* and *Komagataeibacter* genera. Well-known niches of AAB are fruit microflora and kombucha beverage [12]. They are a part of a symbiotic consortium with different yeasts, which are altogether involved in the fermentation process of tea and sucrose during kombucha beverage production [25]. In this research, the isolate from kombucha beverage, with the best yield of BC, was selected for further PCR molecular identification. According to 16s rDNA gene sequencing, it was determined that the best cellulose-producing AAB belong to the *Komagataeibacter rhaeticus* species, showing a similarity of 94.64% with *K. rhaeticus* strains (accession numbers: LC108743.1, CP050139.1, MT093922.1, MT093933.1, and MT093834.1). The GenBank accession number ON680934 for the obtained nucleotide sequence of the isolate was provided. It has been previously shown that *K. rhaeticus* is usually present in kombucha beverages [13,26].

After 4 days of BC production by isolated *K. rhaeticus* in 50 mL of YPM broth, 116.1 ± 3.3 mg of dry weight BC was produced, while extended cultivation processes up to 7 days increased the weight to 145.6 ± 2.0 mg. These yields indicated that this *K. rhaeticus* strain possessed good capacity for BC production. Higher weights were reached after 4 days of production compared to *K. rhaeticus* TJPU03, while the values for dry weights were similar when longer cultivation was applied, i.e., 7 days [12].

After BC modification with HAp/TiO_2_ ceramic material, their incorporation influenced the increase in the final weights, and these results are presented in Table 1. The difference between composites weights and pure BC indicated the amount of incorporated ceramics. Incorporation in 4-day-old BC led to an increase in weight by 50.6 ± 0.8 mg and 30.4 ± 2.9 mg in samples 4I and 4II, respectively. Incorporation of ceramic material in 7-day-old BC increased the weight by 48.9 ± 1.8 mg and 53.8 ± 3.1 mg in samples 7I and 7II, respectively. The significant difference in HAp/TiO_2_ incorporation was noted between samples 4II and 7II that were produced by the second method in the presence of 4-day-old and 7-day-old BCs, respectively. The presence of more negatively charged OH groups in BC cultivated for a longer period, i.e., in 7 days (sample 7K) rather than in 4 days (sample 4K), possibly led to a greater ionic interaction with Ca^2+^ ions, which further bind phosphate ions and form a greater number of initial apatite nuclei. After these nuclei were formed, they grew by calcium and phosphate uptake [27,28], and the weight of the composites increased.

### 3.2. Characterization of BC/HAp/TiO_2_ Composites

#### 3.2.1. Scanning Electron Microscopy (SEM) and Energy-Dispersive X-ray (EDS)

**Figure 2:**
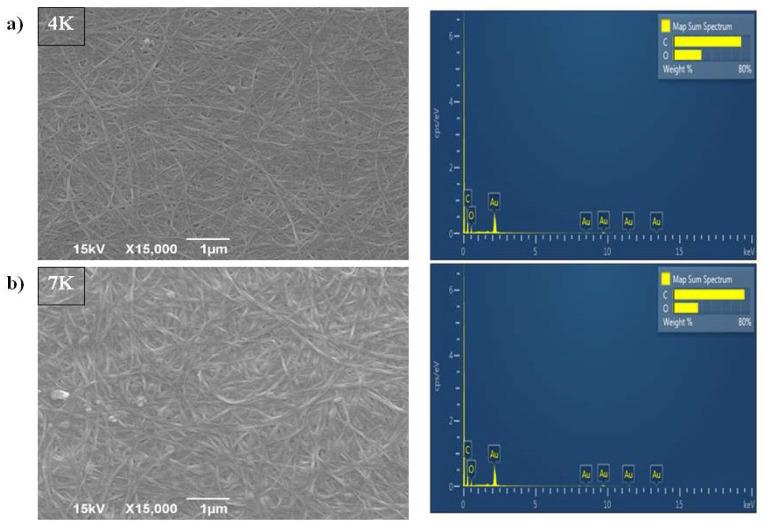
SEM and EDS analysis of pure BC cultivated for (**a**) 4 days (4K); (**b**) 7 days (7K).

Morphology evaluation of the control samples using SEM (Figure 2a,b) showed fibers of pure bacterial cellulose samples, i.e., 4K and 7K, representing a nanosized densely intertwined matrix. Due to an extended biosynthesis process, the 7K sample had a larger diameter of fibers (69.7 ± 15.7 nm) compared to the 4K sample (51.4 ± 13.4 nm). EDS analysis (Figure 2a,b) showed that only C and O elements were constituents of those matrixes, as was expected for pure cellulose. 

**Figure 3:**
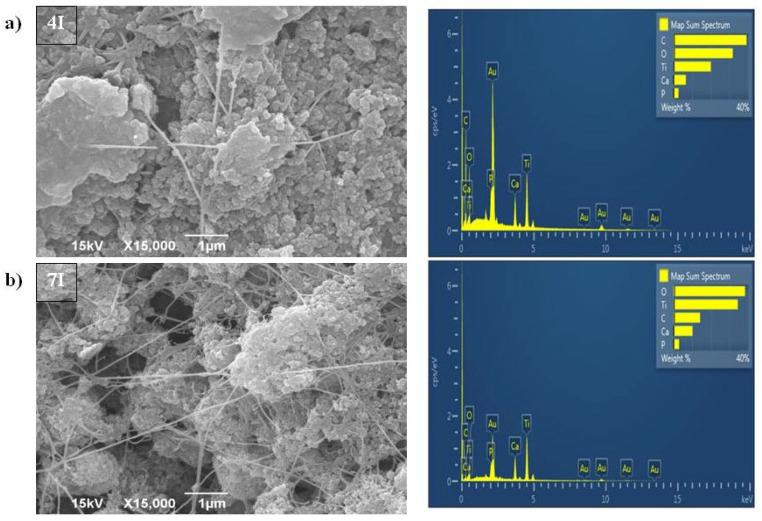
SEM and EDS analysis of BC/HAp/TiO_2_ composite synthesized by the first method for functionalization of BC that was cultivated for (**a**) 4 days (4I) and (**b**) 7 days (7I).

After the BC/HAp/TiO_2_ composite was synthesized by the first method (samples marked as 4I and 7I, and presented in Figure 3a,b, respectively), spherical particles with an average size under 100 nm were recorded. Those particles made agglomerates of up to 2.5 µm. The process of HAp/TiO_2_ introduction into the BC hydrogels led to their pore enlargement. EDS analysis, presented in Figure 3a,b, confirmed the presence of Ca, P, and Ti elements in those samples, as well as C and O elements that were previously found (Figure 2).

**Figure 4:**
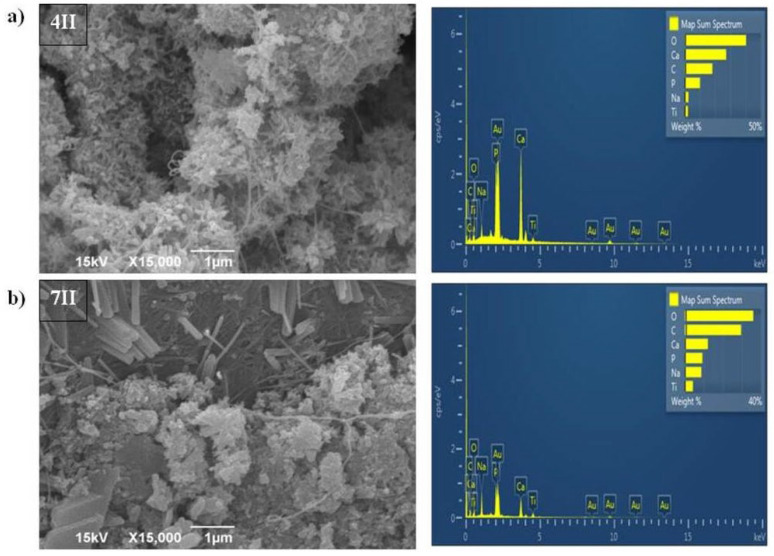
SEM and EDS analysis of BC/HAp/TiO_2_ composite synthesized by the second method for functionalization of BC that was cultivated for (**a**) 4 days (4II) and (**b**) 7 days (7II).

Samples of BC/HAp/TiO_2_ produced by the second method (samples marked as 4II and 7II) had different appearances of the filler particles (Figure 4a,b). They were elongated, rod-shaped, with a size up to 200 nm and were aggregated as well. Sample 7II was less uniform than 4II due to larger rod-shaped crystals, which probably originated from HAp. EDS analysis (Figure 4a,b) confirmed the presence of Ca, P, and Ti elements in samples 4II and 7II. Occurrence of Au element in the samples (Figure 2, Figure 3 and Figure 4) originates from the coating that was applied prior to recording, as described in the Section 2.3. The contents of Ca and P in HAp for samples 4II and 7II were higher than in samples from the first synthesis method, i.e., 4I and 7I. However, the percentages of Ti element in samples 4II and 7II were lower than in the 4I and 7I samples. Moreover, the ultrasound treatment during the first synthesis method disintegrated the BC fiber structure, especially in sample 7I, where a decrease in fiber thickness was observed after modification.

#### 3.2.2. XRD Analysis

**Figure 5:**
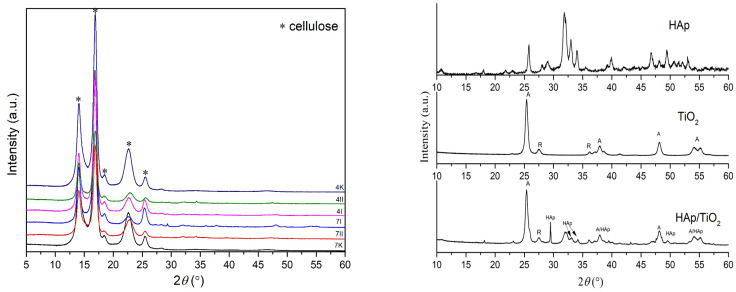
XRD patterns of pure BC, BC/HAp/TiO_2_ composites, HAp, TiO_2_, and HAp/TiO_2_ composite. Control samples of pure BC after 4 (4K) and 7 (7K) days of BC cultivation; synthesized BC/HAp/TiO_2_ composites by the first method after 4 days (4I) and 7 days (7I) of BC cultivation and the second method after 4 days (4II) and 7 days (7II) of BC cultivation; HAp-hydroxyapatite; HAp/TiO_2_-hydroxiapatite/TiO_2_ composite. Note: HAp/TiO_2_ diffractogram was previously published [4] and reproduced by the authors’ permission.

XRD analysis was used to determine a phase composition of the obtained samples, shown in Figure 5. All peaks identified belong to cellulose, where the best match showed with the card number 00-056-1718 ICDD (PDF-2/Release 2012 RDB) [29] belonged to monoclinic space group 4: *P*12_1_1. Additionally, narrow and well-defined peaks indicated that the cellulose had proper structural order as well. A polymer’s degree of crystallinity refers to the order of polymer chains; this is indicated by the overall crystallinity of the phase by taking into account the strongest peaks. Domains in a structure with some degree of stereo regularity were required to determine the degree of crystallinity. For sample 4K, the calculated degree of crystallinity was found to be 79.47%, while for the 7K sample, the crystallinity was 76.48%. The degree of crystallinity in BC from *K. rhaeticus* in this research was higher than in BC produced by *Komagataeibacter intermedius* IMBG180 when 62.07% was determined [30], and lower than in BC produced by *Gluconacetobacter xylinus* 53528 when 83% was found [31]. Depending on the composition of media used for BC synthesis, the degree of crystallinity may vary. Ruka et al. [32] found that a variation in the growing media content for the production of BC by *G. xylinus* determines the crystallinity degree, which can range from 50% to 95%. Based on main reflection intensities of cellulose at about 14.03°, 16.8°, and 22.7° 2*θ*, it was evident that with the addition of HAp and TiO_2_ in the structure, these reflections spread, and their intensity decreased. This implies that HAp and TiO_2_ were successfully incorporated between the cellulose fibers, where it can be said that better results were achieved by the direct method (the second method) of precipitation, especially for sample 4II. Also, with structural changes, i.e., adding HAp and TiO_2_ in the structure, the peaks shifted slightly to the left, which can indicate the interstitial incorporation of HAp/TiO_2_ into cellulose polymer.

Due to peaks overlapping in XRD diffractograms, further clarification of HAp/TiO_2_ incorporation into BC was performed by FTIR analyses.

#### 3.2.3. FTIR Analysis

**Figure 6:**
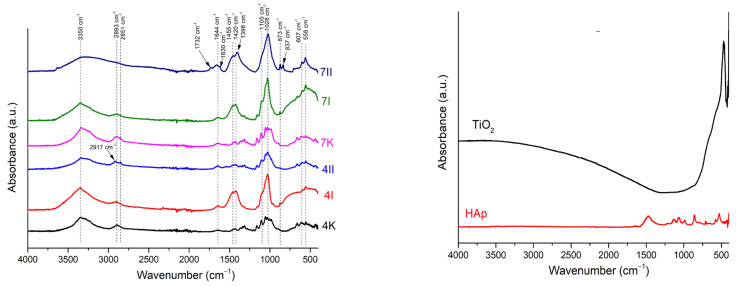
FTIR spectra of (**left**) pure BC and BC/HAp/TiO_2_ composites and (**right**) TiO_2_ and HAp. I: The first method of synthesized BC/HAp/TiO_2_ composite; II: the second method of BC/HAp/TiO_2_ composite synthesis; K: control samples; 4: 4-day-old BC; 7: 7-day-old BC.

The effect of BC modification by ceramic components was followed by FTIR spectroscopy, and the collected spectra are shown in Figure 6.

In all spectra, characteristic absorption bends of pure BC were detected. At a wavenumber of ~3350 cm^−1^, it can be noticed that the absorption bend corresponded from the stretching vibration of the –OH group. Also, the absorption bends originated from the stretching vibrations of the –CH_2_ and –CH_3_ functional groups at ~2900 cm^−1^, and bending vibrations from the absorbed water of the –OH group at ~1644 cm^−1^ were present. The series of absorption bends detected between 1500 cm^−1^ and 1000 cm^−1^, usually present in the spectra of bacterial cellulose obtained from the bacteria *Komagataeibacter* sp. [33], indicated the presence of pure cellulose.

In the spectra of the modified samples, absorption bends corresponding to the vibrations of the distinct groups for TiO_2_ and HAp phases were detected. A broad absorption bend between 450 cm^−1^ and 800 cm^−1^ originated from the stretching vibration in Ti-O-Ti [4,34], which appeared in the spectra labeled as 4I, 7I, and 7II. Although titanium was detected by EDS analysis (Figure 4a), the absence of these peaks in spectra 4II is due to the non-uniform distribution of the ceramic filler in the BC matrix. Besides this broad band, a few sharp absorption bends in this region were detected at the following positions, 559 cm^−1^ and 598 cm^−1^; 966 cm^−1^; 1024 cm^−1^ and 1102 cm^−1^, all attributed to specific vibrations of HAp. All the band positions agreed with the literature [4,7,35,36]. Besides the absorption bends characteristic for PO_4_^3−^ vibrations, the absorption bend positioned at 873 cm^−1^ was observed in all spectra, which corresponded to the vibration in CO_3_^2−^. This carbonate vibration indicated that the partially carbonated apatite was formed, known as a biological, B-type HAp [34]. Carbonate that occurred in samples was probably absorbed from the atmosphere. Furthermore, a shift of the absorption bends in the 1400–1700 cm^−1^ range was noticed for the composite samples compared to pure BC. This shift and broadening of the absorption bends at 1640 cm^−1^ and 1420 cm^−1^ implied the formation of hydrogen bonds between BC and ceramic components [37]. This indicated that the incorporation of the filler particles in the BC matrix affected the vibrations of the BC matrix. The most pronounced shift was obtained for composite 7II, and the lowest was obtained for 4II. In addition, the sample denoted as 7II also showed the highest content of incorporated ceramic filler (Table 1). As previously mentioned, due to non-uniform ceramic component distribution, the spectrum of the 4II sample was collected from the non-modified part of the film. However, based on the results obtained by EDS and SEM analysis (Section 3.2.1), it was evident that HAp and TiO_2_ were incorporated between cellulose fibers.

#### 3.2.4. Mechanical Characterization

The mechanical properties of the produced composites were determined by a tensile test and are shown in Table 2. More prolonged cultivation of pure BC (sample 7K), as well as composites (samples 7I and 7II), caused an increase in BC fiber thickness and mechanical properties, e.g., 19.27% and 29.86% increase in tensile strength and Young’s modulus, respectively, after 7 days of BC cultivation (4K and 7K). However, the mechanical properties and Young’s modulus decreased after the introduction of HAp/TiO_2_ into BC compared with pure BC. The negative impact of the HAp/TiO_2_ incorporation on the mechanical properties is due to weakened hydrogen bonding between cellulose nanofibers that are blocked to contribute to mechanical strength and elasticity [14]. Moreover, the usage of the second method (samples 4II and 7II) of HAp/TiO_2_ incorporation in BC gave more mechanically stable material, which is indicated via higher tensile strength and elongation at the break compared with the usage of the first method (samples 4I and 7I). The trend in decreasing the mechanical properties after the first modification method could be explained by the structural disintegration of the BC fibers after ultrasound dispersion of the pre-prepared HAp/TiO_2_ particles in the BC matrix (Figure 3). This phenomenon is dominant compared to the effect of the size of the incorporated HAp/TiO_2_ particles/agglomerates on the mechanical properties. Nevertheless, the values of Young’s modulus of the samples are still higher than in other research [14,38].

#### 3.2.5. Thermogravimetric Analysis (TGA)

**Figure 7:**
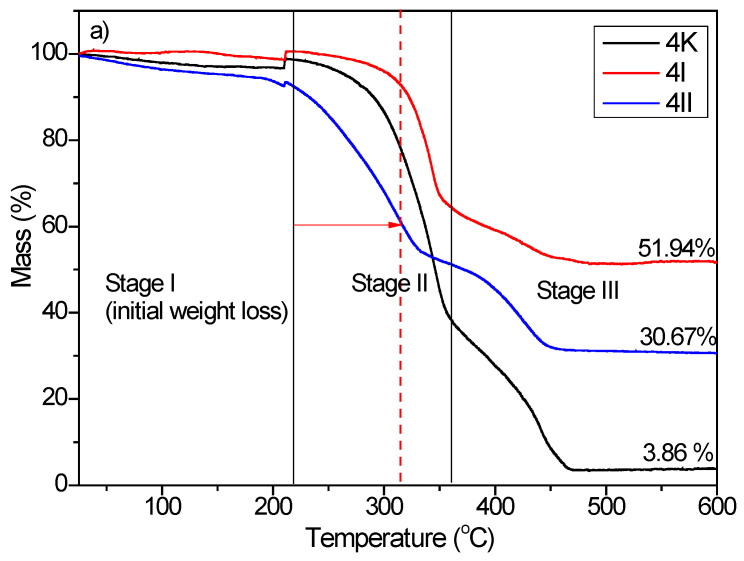

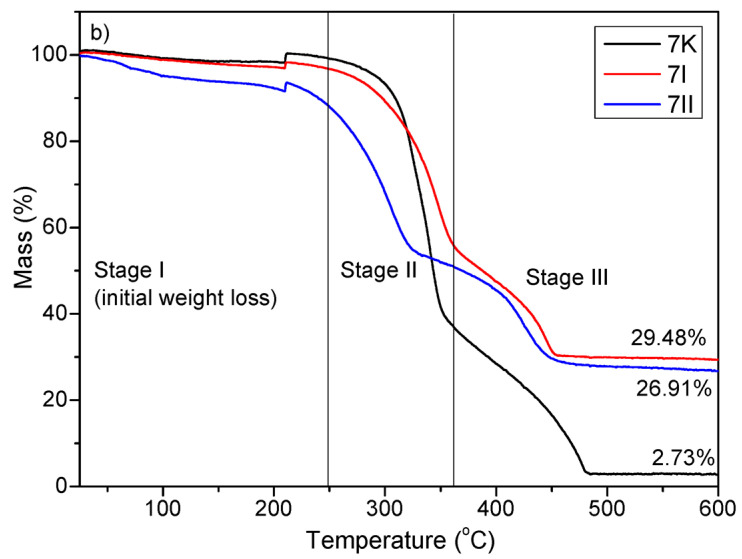
TGA curves of (**a**) 4K, 4I, and 4II and (**b**) 7K, 7I, and 7II composites. 4: four-day-old BC; 7: seven-day-old BC; K: samples with pure BC; I: the first method of synthesized BC/HAp/TiO_2_ composite; II: the second method of synthesized BC/HAp/TiO_2_ composite.

The thermal degradation of the control sample of BC composites (4K and 7K) and TiO_2_/Hap composites also (4I, 4II, 7I, and 7II) took place in three stages of weight loss [39,40]. The obtained TGA curves are shown in Figure 7. Stage I describes the processes which occurred at a decomposition temperature ≤220 °C. The weight loss that occurred in stage I was attributed to water/moisture evaporation/dehydration [40]. For stage II, processes of thermal decomposition of bacterial cellulose occurred between 220 and 360 °C. Levoglucose, combustible volatiles such as C, CO, CO_2_, and H_2_O were produced as a result of thermal degradation of the bacterial cellulose [39,40]. The last stage observed at temperatures higher than 360 °C was followed by the weight loss caused by the carbonization process of the bacterial cellulose.

It should be noted that the residual weight loss of HAp/TiO_2_ composites was higher compared to the control sample (4K—residual weight loss 3.86%; 7K—residual weight loss 2.73%) and amounted to 51.94% and 30.67% for 4I for 4II, respectively, and 29.48% and 26.91% for 7I for 7II, respectively, indicating that the thermal stability of HAp/TiO_2_ composites was significantly improved. According to the obtained results, it can be concluded that prolonged synthesis of the BC composites reduced thermal stability (Figure 7b). This phenomenon occurred due to the higher contribution of organic material (increased BC fiber thickness) in degradation processes.

Moreover, the initial decomposition temperature of the 4I sample was shifted to a higher temperature, indicating a more significant contribution of the first functionalization method to the thermal stability of the bacterial nanocellulose.

#### 3.2.6. Contact Angle

**Figure 8:**
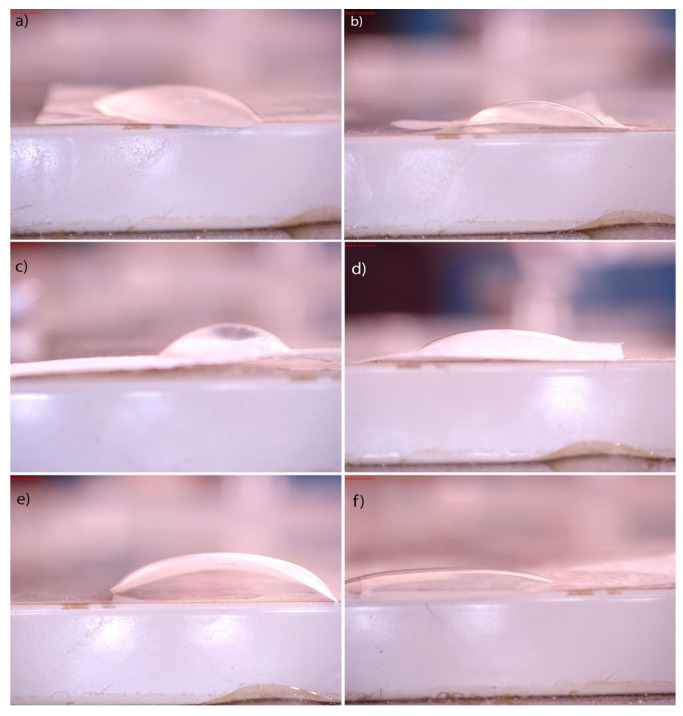
Water contact angles of BC (**a**) and (**b**) and its composites (**c**–**f**). Control samples of pure BC after (**a**) 4 (4K) and (**b**) 7 (7K) days of BC cultivation; synthesized BC/HAp/TiO_2_ composites by the first method after (**c**) 4 days (4I) and (**d**) 7 days (7I) of BC cultivation; the second method after (**e**) 4 days (4II) and (**f**) 7 days (7II) of BC cultivation.

The contact angle results, presented in Table 3 and Figure 8, showed contact angles less than 90° for both groups of BC hydrogels, indicating that the surfaces were favorable for wetting. However, there were apparent differences between BC samples produced in 4 and 7 days. It can be seen that as the period of BC growth increased, the wettability decreased. The functionalization of the 4K sample caused insignificant changes in wettability (≈2.5%). In contrast, the changes in wettability observed in the case of both functionalization procedures of the 7K sample are significantly higher. Water absorption on the film surface can produce more hydroxyl radicals (OH•), which can further react in a photocatalytic reaction and decompose organic matter [41].

### 3.3. Antimicrobial Activity

The results of the antimicrobial activity of the obtained samples are presented in Table 3. The control BC samples (4K and 7K) did not express a statistically significant difference in microbial cell numbers compared to the positive control. This finding is in accordance with previous research that also proved the absence of the antimicrobial activity of pure BC [42]. In comparison to the positive controls, the first method of BC/HAp/TiO_2_ synthesis (4I and 7I samples) did not express a significant reduction in bacterial cells. The only effect was found on *C. albicans* cells for sample 7I, which reduced the cells number by 1.53 log_10_ CFU/mL. Compared to the first method, the second synthesis method proved to be better for synthesizing BC/HAp/TiO_2,_ with both antifungal and antibacterial activity (Table 4). Namely, in the presence of 4II and 7II samples, the number of *C. albicans* yeast cells was reduced to 3.67 log_10_ CFU/mL and 3.39 log_10_ CFU/mL, respectively. Compared to the positive controls, sample 4II reduced the number of *S. aureus* cells to 4.57 log_10_ CFU/mL and *E. coli* cells for 6.20 log_10_ CFU/mL and was the only sample that reduced the number of *P. mirabilis* cells to 1.53 log_10_ CFU/mL (Table 3). In addition, sample 4II acted microbistatically in comparison to inoculum due to a significant reduction in microbial cells, i.e., 53.7% of *C. albicans* (from 4.43 log_10_ CFU/mL to 4.10 log_10_ CFU/mL), 98.11% of *S. aureus* (from 5.02 log_10_ CFU/mL to 4.30 log_10_ CFU/mL), and 99.71% of *E. coli* (from 5.64 log_10_ CFU/mL to 2.48 log_10_ CFU/mL).

According to EDS analysis (Section 3.2.1), when the second synthesis method was used, the obtained samples (4II and 7II) contained higher quantities of Ca and P originating from HAp phase in comparison to samples obtained using the first synthesis method (4I and 7I). Also, the share of Ti in the second method was lower than when the first method was applied (Figure 3 and Figure 4, respectively). These findings indicate the importance of HAp for the better antimicrobial activity of the samples produced by the second method. Composite-based photocatalysts with HAp were shown to act synergistically with TiO_2_ particles and increase their efficiency due to the high specific surface area [7]. Furthermore, it was shown that after the absorption of electromagnetic radiation, the photocatalytic reaction occurred in TiO_2,_ and the electron was excited from the valence band. Excited negatively charged electrons and positively charged valence band holes were involved in reactions of oxidation and reduction with species that come into contact with TiO_2_ surface. Through these reactions, non-selective highly potent hydroxyl radicals (OH•) were produced, which further reacted and decomposed organic substances, pollutants, or microorganisms through a cell wall and DNA destruction, respectively [43].

The weights of incorporated HAp/TiO_2_ in BCs (Table 1) indicate that the amounts of incorporated materials are not crucial to reach antimicrobial activity due to the lowest incorporated amounts in the 4II sample which expressed the best properties (Table 4). It was previously shown that the activity of the composites depended on particle shape, size, and size distribution [2,7]. According to SEM micrographs, when the second method of HAp/TiO_2_ incorporation in the BC matrix was applied (4II and 7II samples), HAp/TiO_2_ crystals were developed in rod-like shapes (Figure 4). This shape might have an important impact on antimicrobial activity due to better results being obtained in comparison with the spherical HAp/TiO_2_ particles obtained by the first method (Figure 3). In addition, smaller crystals were obtained during the synthesis process of the 4II sample, which was the probable reason of higher impact on better antibacterial activity against *S. aureus*, *E. coli,* and *P. mirabilis* than the 7II sample. It was previously shown that smaller-sized nanoparticles as well as rod- or wire-shaped metal oxides (ZnO) pass through the bacterial cell wall more easily than nanomaterials with spherical shapes [2,44]. The larger crystals in sample 7II compared to 4II did not influence activity against eukaryotic cells, i.e., the yeast *C. albicans*. These cells are larger than procaryotic bacterial cells, and bigger crystals were able to penetrate into them.

## 4. Conclusions

In this research, two functionalization methods were applied for the development of advanced BC-based functional polymer–ceramic material. Isolated *K. rhaeticus* species were shown to produce pure, nanosized, and densely intertwined BC polymer with high crystallinity after 4 and 7 days of polymer synthesis. The application of two different methods for HAp/TiO_2_ incorporation into BC resulted in different particle morphologies and compositions. Namely, HAp/TiO_2_ particles were spherical when the first incorporation method into BC matrixes was applied, while after the second method was applied, particles were elongated and rod-shaped. EDS analysis showed that after the application of the second method, the share of Ca and P elements was higher, while the share of Ti elements was lower than after the employment of the first method. Both routes of HAp/TiO_2_ incorporation into the BC polymer did not influence crystallinity. FTIR analysis showed that all characteristic peaks for BC, HAp, and TiO_2_ were detected, proving their successful incorporation. Shorter BC cultivation time and incorporation of the HAp/TiO_2_ particles caused a decrease in mechanical properties due to changes in/disintegration of the BC fibers’ structure and a decrease in the hydrogen bonding/interaction between BC fibers. This is unlike the prolonged synthesis of BC, which reduced thermal stability due to the larger contribution of organic material. The incorporation of HAp/TiO_2_ into BC improved the thermal stability of the composites. As expected, the antimicrobial activity was not found for pure BCs, but it was achieved with HAp/TiO_2_ incorporation. The second method of HAp/TiO_2_ particles integration was found to be a better way to reach the antimicrobial properties of BC, which were dependent on the shape, size, and composition of the particles, but not on the amounts of incorporated materials. Namely, both samples from the second route (4II and 7II) expressed antifungal activity against *C. albicans*, while the 4II sample also significantly reduced the number of bacteria (*S. aureus*, *E. coli,* and *P. mirabilis*). Considering all the previously mentioned findings, in the development of novel functional antimicrobial materials with HAp/TiO_2_ ceramic composites and BC as a carrier polymer, the method of their incorporation into BC is crucial for their activity when incorporated. Furthermore, BC that was biosynthesized for a shorter period, i.e., 4 days, is a more cost-effective option than a 7-day biosynthesis process. Due to the obtained excellent antimicrobial properties, the developed advanced polymer–ceramic material might be proposed for further application as antimicrobial material. Further research will be directed towards the assessment of the durability, wear resistance, and maintenance of antimicrobial activity over an extended timeframe for an advanced understanding of the developed composite material. These additional investigations would contribute valuable insights into the long-term performance and stability of the composite materials, allowing for a deeper understanding and possibilities of potential application in touch surfaces, coatings, and clothing uniforms, especially in environments suitable for spreading diseases, such as in hospitals, to be obtained.

## Figures and Tables

**Table 1:** Weights of produced BCs and their composites.

Sample	Dry Weight (mg/50 mL)	Incorporated HAp/TiO_2_ (mg)
4K	116.1 ± 3.3	
4I	166.7 ± 4.1	50.6 ± 0.8
4II	146.5 ± 6.2	30.4 ± 2.9
7K	145.6 ± 2.0	
7I	194.5 ± 3.8	48.9 ± 1.8
7II	199.4 ± 5.1	53.8 ± 3.1

I: The first method of synthesized BC/HAp/TiO_2_ composite; II: the second method of synthesized BC/HAp/TiO_2_ composite; K: Control samples; 4: 4-day-old BC; 7: 7-day-old BC.

**Table 2:** Mechanical properties of pure BCs and BC/HAp/TiO_2_ composites.

Sample	Tensile Strength σ_m (_MPa)	Elongation at Break ε_m_ (%)	Young’s Modulus E (MPa)
4K	28.75 ± 2.55	3.98 ± 0.22	1639.55 ± 61.22
4I	3.55 ± 0.30	1.97 ± 0.16	165.75 ± 11.31
4II	5.77 ± 0.40	6.09 ± 0.44	167.77 ± 10.58
7K	34.29 ± 2.12	6.24 ± 0.50	2129.14 ± 149.18
7I	8.45 ± 0.91	4.63 ± 0.47	771.42 ± 58.14
7II	12.47 ± 1.15	5.04 ± 0.33	570.27 ± 23.37

K: samples with pure BC; 4: four-day-old BC; 7: seven-day-old BC; I: the first method of synthesized BC/HAp/TiO_2_ composite; II: the second method of synthesized BC/HAp/TiO_2_ composite.

**Table 3:** Water contact angles of BCs and their composites.

Sample	Value Measurement Angle (°)
4K	35.85
7K	44.65
4I	36.73
7I	32.11
4II	34.92
7II	22.79

Control samples of pure BC after 4 (4K) and 7 (7K) days of BC cultivation; synthesized BC/HAp/TiO_2_ composites by the first method after 4 days (4I) and 7 days (7I) of BC cultivation; the second method after 4 days (4II) and 7 days (7II) of BC cultivation.

**Table 4:** The antimicrobial effect and reduction levels expressed in log_10_ CFU/mL of pure BC and BC/HAp/TiO_2_ composites.

Sample	*Candida albicans* ATCC 10231		*Staphylococcus aureus* ATCC 25923		*Escherichia coli* ATCC 25922		*Proteus mirabilis* ATCC 12453	
		Reduction		Reduction		Reduction		Reduction
Positive control ^1^	7.77 ± 0.1 ^a,2^		8.87 ± 0.16 ^a^		8.68 ± 0.08 ^a^		9.61 ± 0.11 ^a^	
4K	7.61 ± 0.13 ^a^	0.16 ± 0.03 ^a^	8.87 ± 0.03 ^a^	0.00 ± 0.13 ^a^	8.53 ± 0.03 ^a^	0.15 ± 0.05 ^a^	9.59 ± 0.01 ^a^	0.02 ± 0.08 ^a^
4I	7.53 ± 0.30 ^a^	0.24 ± 0.04 ^a^	8.67 ± 0.07 ^a^	0.21 ± 0.08 ^a^	8.57 ± 0.11 ^a^	0.11 ± 0.03 ^a^	9.50 ± 0.07 ^a^	0.11 ± 0.04 ^a^
4II	4.10 ± 0.28 ^b^	3.67 ± 0.21 ^b^	4.30 ± 0.12 ^b^	4.57 ± 0.04 ^b^	2.48 ± 0.16 ^b^	6.20 ± 0.08 ^b^	8.08 ± 0.03 ^b^	1.53 ± 0.08 ^b^
7K	7.50 ± 0.14 ^a^	0.27 ± 0.04 ^a^	8.86 ± 0.01 ^a^	0.01 ± 0.03 ^a^	8.54 ± 0.13 ^a^	0.14 ± 0.05 ^a^	9.61 ± 0.01 ^a^	0.00 ± 0.01 ^a^
7I	6.24 ± 0.08 ^c^	1.53 ± 0.02 ^c^	8.80 ± 0.04 ^a^	0.07 ± 0.12 ^a^	8.36 ± 0.03 ^a^	0.32 ± 0.05 ^a^	9.58 ± 0.14 ^a^	0.03 ± 0.03 ^a^
7II	4.38 ± 0.01 ^b^	3.39 ± 0.09 ^b^	8.78 ± 0.07 ^a^	0.09 ± 0.09 ^a^	7.65 ± 0.17 ^c^	1.03 ± 0.01 ^c^	9.57 ± 0.00 ^a^	0.04 ± 0.09 ^a^

^1^ Positive control: samples without BC or composite; K: samples with pure BC; I: the first method of synthesized BC/HAp/TiO_2_ composite; II: the second method of synthesized BC/HAp/TiO_2_ composite; 4: four-day-old BC; 7: seven-day-old BC; ^2^ in the same column, values marked with different letters are significantly different, *p* ≤ 0.05, ANOVA, Tukey’s HSD.

## Data Availability

Data are contained within the article.
